# Characterising the Analgesic Effect of Different Targets for Deep Brain Stimulation in Trigeminal Anaesthesia Dolorosa

**DOI:** 10.1159/000446608

**Published:** 2016-06-21

**Authors:** Hugh P. Sims-Williams, Shazia Javed, Anthony E. Pickering, Nikunj K. Patel

**Affiliations:** ^a^North Bristol NHS Trust, Southmead Hospital, University of Bristol, Bristol, UK; ^b^School of Physiology, Pharmacology and Neuroscience, University of Bristol, Bristol, UK

**Keywords:** De-afferentation pain, Periaqueductal grey, Centromedian-parafascicular nucleus, Quantitative sensory testing, Depth electrode

## Abstract

**Background:**

Several deep brain stimulation (DBS) targets have been explored for the alleviation of trigeminal anaesthesia dolorosa. We aimed to characterise the analgesia produced from the periaqueductal grey (PAG) and centromedian-parafascicular (CmPf) nucleus using a within-subject design.

**Method:**

We report a case series of 3 subjects implanted with PAG and CmPf DBS systems for the treatment of anaesthesia dolorosa. At follow-up, testing of onset and offset times, magnitude, and thermal and mechanical sensitivity was performed.

**Results:**

The mean pain score of the cohort was acutely reduced by 56% (p < 0.05) with PAG and 67% (p < 0.01) with CmPf stimulation at mean time intervals of 38 and 16 min, respectively. The onset time was 12.5 min (p < 0.05) for PAG stimulation and 2.5 min (p < 0.01) for CmPf. The offset time was 2.5 min (p < 0.05) for PAG and 12.5 min (p < 0.01) for CmPf. The two targets were effective at different stimulation frequencies and were not antagonistic in effect.

**Conclusion:**

The mechanisms by which stimulation at these two targets produces analgesia are likely to be different. Certain pain qualities may respond more favourably to specific targets. Knowledge of onset and offset times for the targets can guide optimisation of stimulation settings. The use of more than one stimulation target may be beneficial and should be considered in anaesthesia dolorosa patients.

## Introduction

Several deep brain stimulation (DBS) targets have been described for the alleviation of resistant central neuropathic pain syndromes, including the periaqueductal grey (PAG) [1], centromedian-parafascicular (CmPf) nucleus [2], sensory thalamus [3], subthalamic nucleus [4], nucleus accumbens [5], and anterior cingulate cortex [6]. These activate different neuronal pathways and are likely to alter pain perception by distinct mechanisms. There have been no studies to date comparing the acute analgesic effects of stimulation at the PAG and CmPf targets or the consequences of combined site stimulation.

PAG stimulation is thought (in part) to exert its actions by a release of endogenous opioid [7,8] via a descending modulatory pathway. Low-frequency stimulation of PAG results in a sensation of warmth, pleasure, satiety, and inebriation [9]. Another target, CmPf, was found serendipitously (after the post-mortem determined the exact site of depth electrodes) after successful treatment of a patient with terminal cancer [10]. Despite the close proximity of CmPf to the PAG, anatomical and physiological studies suggest a separate mechanism of analgesia [2]. It is thought to reduce the affective component of pain via ascending connections to the anterior cingulate nucleus [11].

By studying patients with electrodes implanted into these two targets for the successful treatment of trigeminal anaesthesia dolorosa, we attempt to characterise the analgesic effect of each target and assess whether a greater analgesic effect can be achieved through combined stimulation. A meta-analysis of DBS for analgesia found a greater mean reduction in pain where electrodes targeted more than one anatomical site [12]. This group only confirmed that multiple targets increased the likelihood of successful stimulation-induced analgesia, but the effect of each target alone and in combination was not assessed. We hypothesised that PAG and CmPf stimulation would each exhibit distinct characteristics and that simultaneous stimulation may be beneficial. Improved understanding of timing and response to stimulation in these two sites may better inform patient and target selection and assist optimisation of treatment. We report a small case series with characterisation of the analgesic effects of two stimulation sites both individually and in combination.

## Methods

Full approval from the NHS ethics committee (Ref. 12/SW/0255) and the North Bristol NHS Trust research and development (Ref. 2875) was obtained for the testing performed. The study was registered with the National Institute for Health Research Comprehensive Clinical Research Network (Ref. 13580).

### Operative Method

Patients were required to have symptoms and a confirmed mechanism of trigeminal nerve damage to be considered for DBS (see table 3). Patients with no response to optimal conventional treatment under a secondary pain service for 2 years were offered DBS as per criteria of the NHS exceptional clinical need panel.

Planning MRI scans were obtained under general anaesthesia using a Leksell frame and localiser unit. Trajectory and co-ordinates for the ventral PAG and CmPf contralateral to the pain were derived from individual subject MRIs using NeuroInspire™ software (Renishaw, Wotton-under-Edge, UK). Guide tubes were placed using stereotaxy, and the position was confirmed with CT angiogram. The position of the electrodes (Medtronic 3387) could then be modified based on the position of the guide tube. Electrode tips protrude 10 mm beyond the guide tube in the same trajectory, so contact positions could be confirmed using image software [13]. Each electrode was connected to its own impulse generator (IPG) as the targets required different stimulation frequencies. Medtronic Itrel 3 single-channel IPGs were implanted below the clavicle bilaterally (with the right-sided IPG always connected to the PAG electrode to avoid confusion when programming). Programming of IPG was performed 3-5 days after electrode insertion. Repeat programing was carried out at routine outpatient follow-up by the neurosurgical research fellow.

### Method for Characterising Stimulation Effects

**Fig. 1:**
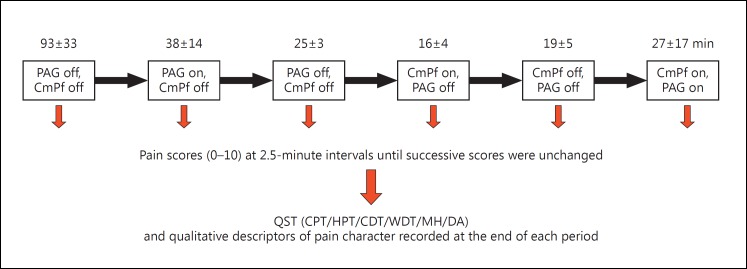
The stimulation testing protocol performed in the clinic after a mean follow-up period of 19 months. CPT = Cold pain threshold; HPT = hot pain threshold, CDT = cold detection threshold; WDT = warm detection threshold; MH = mechanical hypersensitivity; DA = dynamic allodynia. The mean time period to achieve stabilisation of pain scores is provided along with the standard error (SE) to the nearest minute above each section. Stimulation off times had to be individualised to minimise patient discomfort as per ethical approval.

All subjects operated on between 2010 and 2012 for anaesthesia dolorosa were included in the study. No subjects were excluded. All subjects during this period had dual target stimulation. The mean follow-up period between DBS insertion and this study was 19 ± 8 months. Testing for acute analgesic effect, onset/offset of effect, and character of analgesic effect using quantitative sensory testing (QST) were all carried out over a single day using the protocol shown in figure 1.

Each patient had both pulse generators switched off prior to commencing the testing protocol. This was for 60 min or longer until the pain score stabilised (three static readings). Mean timings for each part of the protocol are provided in figure 1. Each section of the testing protocol was continued until the subject's pain score had stabilised, thus the durations on and off stimulation varied between subjects and target used. This was to ensure the minimum period of discomfort experienced by the patient when stimulation was switched off and to reduce the length of the testing protocol where no further analgesic benefit was noted in the ‘stimulation on’ phase.

Numeric rating scores (Numeric Rating Scale) for pain were assessed at 2.5-min intervals during each section of the protocol. A baseline pain score was established prior to the next target being stimulated. Open questions about the pain character, severity, and effect of fan-blown air on the face were asked during each stage.

QST was performed at the end of each section. The subject clicked a button when they could sense a change in temperature or discomfort from the thermode placed on their affected cheek (surface area of 9 cm^2^). The subjects were asked to close their eyes during all testing. A thermal sensory analyser (TSA 2001-II; MEDOC, Israel) was used to determine the cold detection threshold, warm detection threshold, cold pain threshold, and hot pain threshold. The thermode temperature was increased or decreased from 32°C at 1°C per second for temperature detection and 1.5°C per second for temperature pain threshold using the ‘limits method’ [14]. The threshold was determined from the mean of four trials for cold and warm sensory detection [15] and three trials for hot and cold pain thresholds. Mechanical hypersensitivity was assessed using von Frey hairs, and dynamic allodynia was determined by brushing the skin with a cotton wool bud.

Statistical analysis was performed using PRISM software (V6 Graphpad, La Jolla, Calif., USA). A repeated-measures one-way ANOVA test was used to assess the significance of pain scores over time and between different stimulation combinations. Sidak's post hoc multiple comparison test was used with correction for multiple comparisons. Changes in sensory and pain temperature thresholds from PAG stimulation were assessed using paired t tests.

## Results

### Patient Characteristics

The mean age of the cohort was 44.3 ± 6.7 years. Surgery was deemed highly successful in all subjects whose mean pain reduction was 67.3 ± 6.7% at the time of recruitment for this study. This was after a mean duration of 18.7 ± 8.0 months of DBS. The range of stimulation frequencies producing optimal analgesia was 5-10 Hz for the PAG electrode and 70-150 Hz for the CmPf electrode (table 1). A comparison of analgesic medication prior to implantation with that being used at the time of this study has been provided (table 2).

### Quantifying Stimulation-Derived Analgesia

**Fig. 2:**
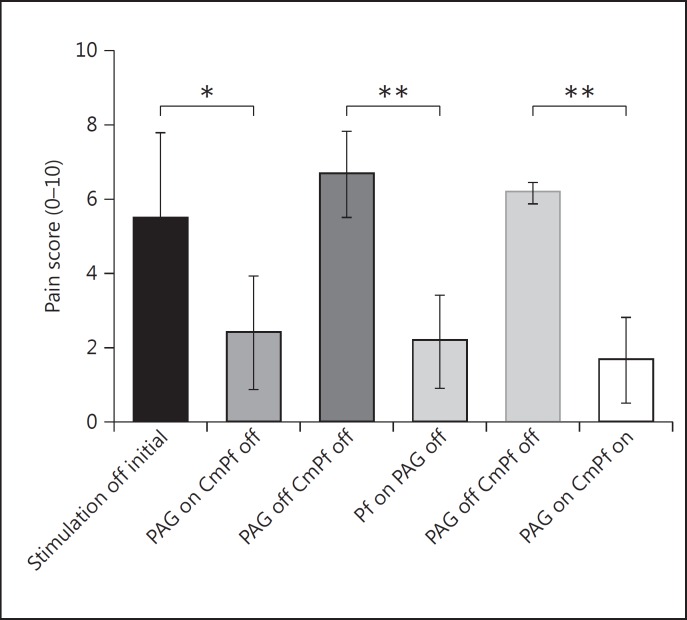
Mean pain score reduction for different DBS sites. * p < 0.05, ** p < 0.01; SE displayed using error bars.

**Fig. 3:**
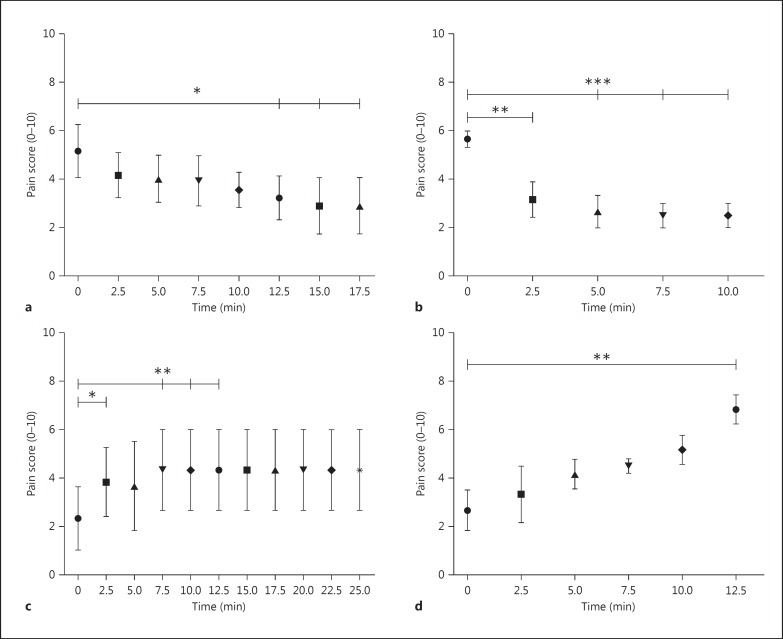
Timing of onset and offset of analgesia in response to DBS at the PAG and CmPf nucleus in anaesthesia dolorosa subjects. **a** Mean pain score against time after PAG stimulation is switched on (p = 0.03, F = 3.1, d.f. = 7). **b** Mean pain score against time after CmPf stimulation is switched on (p = 0.0002, F = 21.9, d.f. = 4). **c** Mean pain score in response to PAG stimulation being stopped (p = 0.01, F = 5.5, d.f. = 5). **d** Mean pain score in response to CmPf stimulation being stopped. (p = 0.01, F = 5.7, d.f. = 5). SE bars are provided at each mean pain score. * p < 0.05, ** p < 0.01, *** p < 0.005, referring to sub-analysis using Sidak's test.

The pain score changed significantly with each stimulation combination (p < 0.05, F score 8.97, 5 d.f.). The mean pain score of the cohort (Numeric Rating Scale) was acutely reduced from 5.5 ± 1.3 to 2.4 ± 0.9 (56% reduction, p < 0.05) for PAG stimulation and from 6.7 ± 0.7 to 2.2 ± 0.7 (67% reduction, p < 0.01) for CmPf stimulation (fig. 2). Dual target stimulation produced a mean reduction in pain score from 6.2 ± 0.2 to 1.7 ± 0.7 (73% reduction, p < 0.01). There was no significant difference between the degrees of analgesia seen with individual or combined stimulation targets in our small sample. The change in pain score was recorded over a testing period of 38 ± 14, 16 ± 4, and 27 ± 17 min for PAG alone, CmPf alone, and both PAG and CmPf stimulation, respectively (fig. 1, 3).

*Onset Time.* The time taken to generate a significant analgesic response to stimulation was found at 12.5 min (p < 0.01) for PAG and 2.5 min for CmPf (p < 0.001; fig. 3a, b).

*Washout.* When stimulation was withdrawn (after having been on for a minimum of 40 min), a significant reversal of the analgesic effect was apparent at 2.5 min (p < 0.01) for PAG stimulation and at 12.5 min (p < 0.01) for CmPf stimulation.

**Fig. 4:**
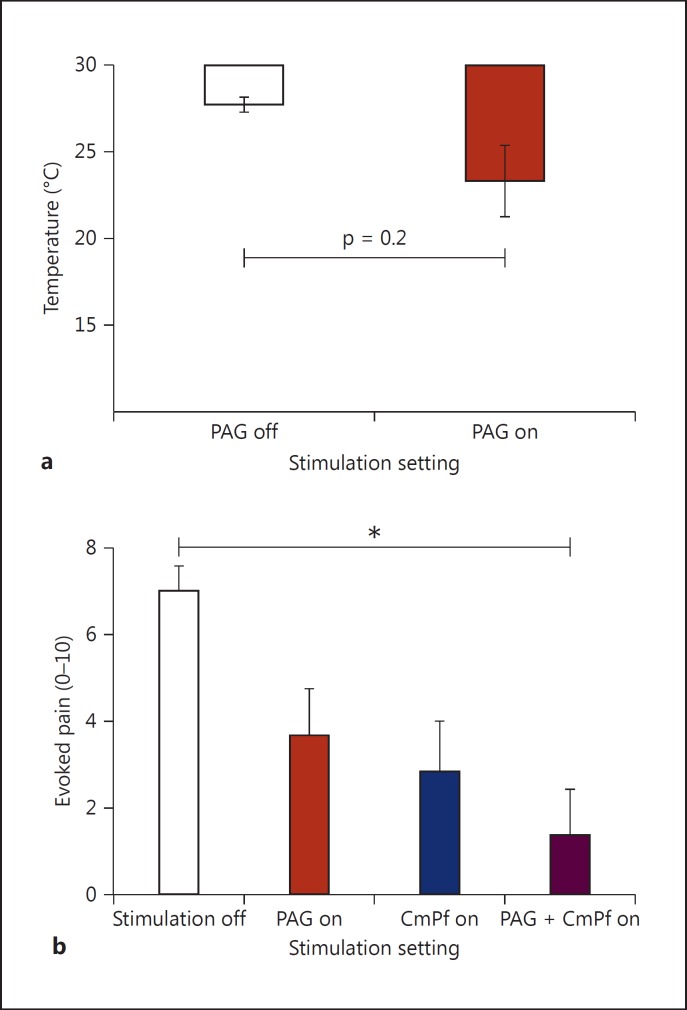
Positive QST findings (timing between stimulation combinations as per figure I). **a** Change in cold pain threshold after a mean of 38 minutes (±14) of PAG stimulation. Two-tailed paired t test (p = 0.2). **b** Change in dynamic allodynia score (Numeric Rating Scale) for each stimulation combination. * p < 0.05.

*Characterising Analgesic Effect through QST.* All 3 subjects demonstrated improvements in their cold pain threshold with PAG stimulation from 27.7 ± 0.4 to 23.3 ± 2.0°C, but the change was not significant (two-tailed paired t test, p = 0.2; fig. 4). No trend was seen in cold pain threshold with CmPf stimulation. Stimulation resulted in no change to other QST temperature thresholds (warm detection threshold, hot pain threshold, cold detection threshold), nor was there a change in mechanical hypersensitivity as assayed with von Frey hairs. However, dynamic allodynia, as tested with a brush from a cotton bud, was attenuated by both PAG and CmPf stimulation. This approached significance (p = 0.058, F = 4.4, d.f. = 3). The post hoc test revealed that when PAG and CmPf stimulation were used in combination there was a significant change in allodynia score (p = 0.04, d.f. = 6; fig. 4).

*Qualitative Characterisation of Analgesic Effect.* Qualitative changes in pain were recorded in response to stimulation at both targets (table 3). PAG stimulation attenuated spasm frequency and provided a sensation of warmth bilaterally. CmPf appeared to reduce the unpleasantness of pain while providing a distracting paraesthesia over the painful area. A summary of all observations is provided (table 4).

## Discussion

### DBS Target Choice

DBS has been employed for chronic pain with varying degrees of success [16]. This may reflect the challenge of target selection in a complex patient population. However, those who do benefit report substantial relief of previously intractable pain [12]. It has been postulated that PAG stimulation is better suited to nociceptive pain, whereas sensory thalamic and potentially CmPf targets are more effective in neuropathic pain [1,2]. Our data also point to different mechanisms for the analgesia produced by PAG and CmPf stimulation. Historically, only subjects responding to a trial of systemic morphine followed by naloxone reversal were offered PAG stimulation [1], but this selection process has not been widely adopted. We propose that characterisation of patients' pain experience may help to direct anti-nociceptive target selection, improving patient outcomes.

### Timing

The onset and offset times and analgesic characteristics for stimulation-derived analgesia have not been examined in the follow-up setting in humans before. Intra-operatively ‘a pleasant warmth’ has been described after 15-20 min of PAG stimulation [1]. PAG stimulation offset times are as short as 5 min in rodents [7] and may be as long as 24 h in man [17]. Even less has been documented concerning CmPf stimulation onset and offset times in humans. We found a reliable time frame for the onset and offset of acute effects in our cohort of 3 anaesthesia dolorosa subjects (which was replicated in our phantom limb pain patients; unpubl. data). Our results should inform clinicians performing intra-operative trials of micro-stimulation and those involved in DBS programming.

### Quantitative Sensory Testing

Several studies suggest that temperature-related or burning pain [18,19] responds well to PAG stimulation. In support of this we have demonstrated that PAG stimulation objectively alters the cold pain threshold using QST. Considering the large changes in the overall pain score it is surprising that changes to thermal and mechanical static pain are so small. This does not support a principal mechanism of anti-nociception via a descending action on nociceptive pathways in general, and other mechanisms of action for analgesia must be inferred.

### Allodynia Attenuation

Attenuated sensitivity to a cotton bud brush, as a measure of tactile allodynia, was demonstrated with both PAG and CmPf stimulation. This reduction in hyperalgesia in the presence of normal static mechanical sensation has been described previously in response to PAG stimulation for post-stroke pain [18].

### Qualitative Aspects of Pain Character

CmPf stimulation appears to modify the affective and motivational components of pain [2]. This was noted by all of our subjects who reported the pain to be less unpleasant. The CmPf is connected directly to the anterior cingulate cortex [19] and indirectly to the amygdala via the medial dorsal nucleus of the thalamus, which may explain its ability to influence these emotional aspects of pain.

### Different Mechanisms

The different frequencies, analgesic qualities, and connectivity seen in PAG and CmPf stimulation strengthen our hypothesis of differing mechanisms and support the use of these targets in combination. Our results suggest that the analgesic effects of PAG and CmPf stimulation may be useful in combination, but we were unable to demonstrate any synergy in their effects on the overall pain score. However, all patients preferred using both pulse generators in combination as each target provided useful qualitatively distinct aspects of relief to their pain. Bittar et al. [12] have described increased success of surgery where two or more targets were implanted. This was thought to be due to the complexity of selecting the correct target for the specific pain type. Our results take this one step further: by using targets which act through different mechanisms we may be able to offer patients a more comprehensive analgesic response to their pain syndrome with no loss of efficacy at either target.

## Conclusion

The mechanisms by which the stimulation targets PAG and CmPf achieve analgesia are likely to be different. Certain pain qualities may favour specific targets. PAG stimulation may be effective against cold pain, whereas CmPf stimulation may alter the affective and motivational components of pain, while providing a distractive paraesthesia. Improved understanding of timing and response to stimulation in these two anatomical locations may better inform patient selection and promote optimal treatment and programming. Pain characteristics in this population are multi-faceted. The effect of using PAG and CmPf targets simultaneously does not result in antagonism and may offer a broader analgesic response to complex pain symptomatology. The use of more than one stimulation target should be considered for patients with trigeminal anaesthesia dolorosa undergoing DBS.

## Figures and Tables

**Table 1:** Characteristics, DBS duration, analgesic response, and stimulation settings for subjects operated on between 2010 and 2012 for anaesthesia dolorosa

Subject ID	Sex	Age, years	Duration of DBS, months	Pain score before surgery	Pain score at 1 week (% reduction)	Pain at most recent clinic visit (% reduction)	PAG settings	CmPf settings
1	F	50	15	9	2 (78)	2 (78)	1 V × 150 μs at 10 Hz (lead 2–, 3–, case+)	2.5 V × 90 μs at 120 Hz (lead 3–, case +)

2	M	31	7	10	3 (70)	4.5 (55)	4.3 V × 90 μs at 10 Hz (lead 0–, 1–, 2, 3, case+)	2 V × 60 μs at 150 Hz (lead 2+, 3-)

3	F	52	34	8.5	0 (100)	3 (69)	5 V × 120 μs at 5 Hz (lead 0–, 3+)	2.5 V × 90 μs at 70 Hz (lead 0–, 1+)

Pain as a function of Numeric Rating Scale pain score, with percentage relief provided in brackets.

**Table 2:** Medication prior to DBS implantation compared to 6-week medication diary performed at the time of this study

Subject	Prior to DBS implantation	At time of study
1	Paracetamol 1 g p.r.n.	Paracetamol 1 g p.r.n.
	Pregabalin 200 mg t.d.s.	Pregabalin 200 mg b.d.
	Venlafaxine M/R 150 mg o.d.	Venlafaxine M/R 150 mg o.d.
	Diazepam 4 mg p.r.n.	Diazepam 4 mg p.r.n.
	Tramadol M/R 150 mg nocte	
	Carbamazepine 100 mg 6× per day	
	(previously: Gabapentin)	

2	Gabapentin 700 mg t.d.s.	No medication
	Buprenorphine 10 μg/h	
	Lignocaine patch 5%	
	Tramadol 100 mg p.r.n.	
	Mirtazapine 30 mg b.d.	
	Nortriptyline 75 mg o.d.	
	(previously: Carbamazepine, Oxycarbamazepine,	
	Lamotrigine, Oxycodone, intravenous/nasal lidocaine)	

3	Morphine (MST) 30 mg b.d.	Morphine (MST) 30 mg o.d.
	Amitriptyline 150 mg nocte	Amitriptyline 150 mg nocte
	Pregabalin 300 b.d.	Pregabalin 150 mg b.d.
	Carbamazepine 200 mg 5× per day	
	(previously: Fluoxetine, Venlafaxine, Sodium Valproate and Gabapentin)	

**Table 3:** Qualitative data showing the effect of stimulation on the character of pain

Subject	Mechanism of injury	Pain quality	PAG effect	CmPf effect
1	Diagnostic glycerol injection into the trigeminal nerve ganglion performed with no relief of symptoms and complicated by a loss of sensation to V1, V2, and V3 dermatomes	Constant burning pain; reduced sensation and paraesthesia over V1, V2, and V3 dermatomes; pain increased by noise	Skin feels less tight; reduction in pins and needles; can tolerate wind on face	Face pain duller; cheek feels the same pain but perceived as less unpleasant

2	Assaulted in 2005, sustaining multiple facial stab wounds from a screwdriver; a fracture through his left zygoma resulted in a unilateral loss of facial sensation and taste; an MRI demonstrated no vascular compression of the trigeminal nerve ganglion, only a cyst-like lesion (which may have been a neuroma) in the left maxillary sinus, which was surgically excised; subsequent to this operation his facial pain worsened acutely	Continuous throbbing, aching pain like something very hot or cold is running across the cheek; paroxysmal shooting pains within the maxillary branch (V2) of the trigeminal nerve; shooting pain triggered by cold air, laughing, talking, and eating; severe allodynia over V2 dermatome	Reduction in frequency of shooting pains and relief from aching pain	Skin feels numb; less sensitive to wind on face; dizzy for 30 s when first switched on

3	Diagnosed with trigeminal neuralgia; MRI confirmed vascular compression, and microvascular decompression alleviated her symptoms for 6 weeks before the pain returned; a partial sensory rhizotomy was performed which qualitatively altered the characteristics of her pain in keeping with the development of anaesthesia dolorosa	Tingling in cheek present 90% of time; burning and shooting pains triggered by radiant heat or cold air, talking, and hot drinks; increased sensitivity with noise	Slight warm sensation across face; reduced sensitivity to cold air	Patient described having a ‘comfortable’ pain: ‘it is still there but I can tolerate it and it doesn't seem to affect me so much’; tingling over face; reduced allodynia to wind/touch

**Table 4:** Summary comparison of the characteristics of stimulation targets individually and in combination

Target	Frequency, Hz	Approximate time for analgesic effect to stabilise with stimulation, min	Approximate time for analgesia reversal to stabilise (washout) with stimulation arrest, min	Analgesic effect	Character of analgesia	Increases threshold to cold pain	Attenuates dynamic allodynia
PAG	5–10	15	7.5	yes	Warmth; feeling of contentment	yes	yes

CmPf	70–150	7.5	12.5	yes	Pain better tolerated; paraesthesia provides distractive paraesthesia	no	yes

Combined				yes	both	yes	yes
